# Genotype–Phenotype Correlations in *PRPH2* Retinopathies: A Comprehensive Analysis of 36 Patients from the Oxford Eye Hospital, UK

**DOI:** 10.3390/genes16091016

**Published:** 2025-08-27

**Authors:** Saoud Al-Khuzaei, Mital Shah, Arun Reginald, Edna Baba, Morag Shanks, Penny Clouston, Robert E. MacLaren, Stephanie Halford, Samantha R. De Silva, Susan M. Downes

**Affiliations:** 1 Nuffield Laboratory of Ophthalmology, Nuffield Department of Clinical Neurosciences, University of Oxford, Oxford OX3 9DU, UKsamantha.desilva@ndcn.ox.ac.uk (S.R.D.S.); 2 Oxford Eye Hospital, Oxford University Hospitals NHS Foundation Trust, Oxford OX3 9DU, UK; 3 The Hospital for Sick Children, University of Toronto, 555 University Avenue, Toronto, ON M5G 1X8, Canada; 4 Oxford Medical Genetics Laboratories, Oxford University Hospitals NHS Foundation Trust, Oxford OX3 7LE, UK; 5 Oxford Centre for Genomic Medicine, Oxford University Hospitals NHS Foundation Trust, Oxford OX3 7HE, UK

**Keywords:** *PRPH2*, pattern dystrophy, genetics, inherited, OCT, fundus autofluorescencec

## Abstract

Purpose: To investigate genotype–phenotype correlations in *PRPH2*-retinopathies in a cohort of 36 patients from the Oxford Eye Hospital and report on novel pathogenic variants. Methods: Clinical data, including best corrected visual acuities (BCVA), fundus autofluorescence (FAF), and optical coherence tomography (OCT) imaging, were analysed. Genetic testing was performed using next-generation sequencing (NGS). Results: In this cohort, 26 different *PRPH2* variants, including 8 novel variants, were identified. Variants were clustered in the D2 loop of the protein. A diverse range of phenotypes were observed: pseudo-Stargardt pattern dystrophy (PSPD) (47.2%), adult-onset vitelliform macular dystrophy (AVMD) (22.2%), pattern dystrophy (PD) (25.0%), atypical macular dystrophy (2.8%), and retinitis pigmentosa (RP) (2.8%). The mean age of symptom onset was 44.0 ± 14.4 years. Mean BCVA was 0.20 ± 0.54 logMAR OD and 0.14 ± 0.29 logMAR OS at baseline and 0.33 ± 0.40 logMAR OD and 0.32 ± 0.40 logMAR OS after a mean follow up duration of 6.0 ± 3.2 years (range 1–11 years). A thickened ellipsoid zone (EZ) was noted in 34/36 patients with a mean EZ thickness of 44.3 ± 11.3 µm OD and 42.7 ± 11.6 µm OS. No clear genotype–phenotype correlations were observed. Conclusions: The significant phenotypic range described in this study is consistent with the previously reported phenotypic variability in *PRPH2* retinopathy and emphasises the complexity of establishing genotype–phenotype correlations in this disease. The thickness of the EZ on OCT may serve as a useful biomarker in distinguishing *PRPH2* retinopathy from other phenocopies. These findings contribute to improved understanding of *PRPH2* retinopathy and help inform diagnosis and genetic counselling.

## 1. Introduction

Inherited retinal diseases (IRDs) encompass a group of genetically determined conditions that often lead to a progressive loss in vision [1]. IRDs are reported to have a prevalence of 1/2500–1/3000 and are now the leading cause of blindness in the working-age population in the United Kingdom [2]. Mutations in over 330 genes have been associated with IRDs (https://retnet.org/, accessed on 28 June 2025). Among these, mutations in the peripherin 2 (*PRPH2*) gene (OMIM: 179605), also known as retinal degeneration slow (RDS) [3,4], are one of the most frequent causes of IRD [5] accounting for 4.6% of IRD cases in the UK [5], 3.4% in Japan [6], and 5% in Italy [7]. However, data on non-European populations remain limited.

The *PRPH2* gene is located on chromosome 6p21.2 and consists of three exons that produce a transcript of 1041 bp [3,4]. *PRPH2* retinopathies are associated with an autosomal dominant inheritance pattern [3,8,9], and incomplete penetrance has been described [3,8,10,11,12,13], as well as significant interfamilial and intrafamilial phenotypic variability [3,14,15]. Moreover, autosomal recessive [16] and digenic inheritance with *ROM1* [17,18] have also been reported.

*PRPH2* encodes a 346-amino acid tetraspanin transmembrane protein containing four transmembrane domains (TMDs), two intradiscal loops (D1 and D2), and N- and C-termini [19,20,21,22]. PRPH2 is expressed in the rims of the photoreceptor outer segments discs and lamellae, playing an essential role in their formation and maintenance [3,23,24,25,26]. The D2 loop contains six cysteine residues (Cys165, Cys166, Cys213, Cys214, Cys222, Cys250) that form disulphide bonds crucial for maintaining the loop structure and regulating photoreceptor folding [26,27,28,29]. Additionally, a seventh unpaired cysteine residue (Cys150) that mediates tetramer polymerisation is present [28]. The D2 loop also facilitates the formation of homo-oligomers and complexes with retinal outer segment membrane protein 1 (ROM1) between Cys165 and Asn182 [3,29,30].

Mouse models have shown that Prph2^−/−^ mice lack outer segments, whilst the Prph2^+/−^ mice have disorganised discs, indicating a dose-dependent variation in phenotype [23,31,32,33].

To date, over 300 variants in *PRPH2* have been reported (https://databases.lovd.nl/shared/genes/PRPH2, accessed on 28 June 2025). Most mutations occur within the D2 loop [3], with studies reporting up to 82% of different *PRPH2* variants affecting this region [34,35,36,37], highlighting its crucial role.

*PRPH2* retinopathy exhibits significant phenotypic variability, and the associated phenotypes include adult vitelliform macular dystrophy (AVMD: OMIM 608161), pattern dystrophy (PD OMIM: 169150), including butterfly-shaped pattern dystrophy, central areolar choroidal dystrophy (CACD: OMIM 613105), pseudo-Stargardt pattern dystrophy (PSPD: OMIM 169150), and retinitis pigmentosa (RP: OMIM 608133) [3,13,34,35,38,39,40,41]. Patients with Leber congenital amaurosis (LCA: OMIM 608133) have been reported with homozygous *PRPH2* mutations [42,43]. Generally, patients with PD/AVMD/PSPD/CACD may be asymptomatic until the fifth decade [3,40], whilst patients with the autosomal dominant RP phenotype usually present in the third to fifth decade [3,17,18]. Choroidal neovascularisation (CNV) has also been reported to occur in some cases of *PRPH2* retinopathy [35,44,45,46,47,48].

The aim of this study is to provide a comprehensive analysis of genotype–phenotype correlation in *PRPH2* in a cohort of patients from the Oxford Eye Hospital. These new data add to the previous literature, further enhancing our understanding of *PRPH2* retinopathies and the potential selection of patients when appropriate gene-targeted therapies become available.

## 2. Materials and Methods

### 2.1. Patient Recruitment 

All patients with at least one variant in *PRPH2* detected between July 2013 and October 2021 by the Oxford University Hospitals Medical Genetics Laboratory were retrospectively identified from an IRD database. Patients with one or more *PRPH2* variants were included if they had a phenotype consistent with PRPH2 retinopathy. Patients with variants in different IRD gene(s) that could explain the phenotype and those with likely benign and benign variants in *PRPH2* were excluded. This study adhered to the tenets of the Declaration of Helsinki and was approved by the Essex 2 Research Ethics Committee (RETGENE 08/H0302/96). Informed consent was obtained from all participants.

### 2.2. Clinical Phenotype

All patients were reviewed in a specialist ophthalmic genetics clinic at the Oxford Eye Hospital, where a detailed medical history was taken and a clinical eye examination was performed. Clinical records were reviewed to collect data on the patients’ age, sex, age of symptom onset, symptoms, family history, any associated conditions, and best corrected visual acuities (BCVAs). The BCVA was recorded either on Snellen or LogMAR charts, and all measurements were converted to LogMAR for the purpose of statistical analysis. A BCVA of counting fingers (CFs) and hand movements (HMs) was converted to 2.5 LogMAR and 3.0 LogMAR, respectively.

Retinal imaging (after pupil dilation with tropicamide 1% and phenylephrine 2.5%) included wide-field retinal imaging (Optomap A10022; Optos Ltd.), short-wavelength fundus autofluorescence (Spectralis; Heidelberg Engineering, Heidelberg, Germany), and spectral-domain optical coherence tomography (SD-OCT) (Spectralis; Heidelberg Engineering, Heidelberg, Germany). Retinal images were assessed by SA-K, SMD, and SRDS. The phenotypic classes used in this study were based on previously reported clinical features. The phenotypes were classified as PD, AVMD, pseudo-Stargardt pattern dystrophy (PSPD), CACD, and RP. PD and AVMD were grouped together for analysis. Eyes with co-pathology were excluded from analysis. Electroretinography, when available, was performed in accordance with the standards of the International Society of Electrophysiology of Vision (ISCEV) using DTL fibre electrodes and an impedance < 5 kOhms in pupils dilated with 1% tropicamide [49].

**Figure 1:**
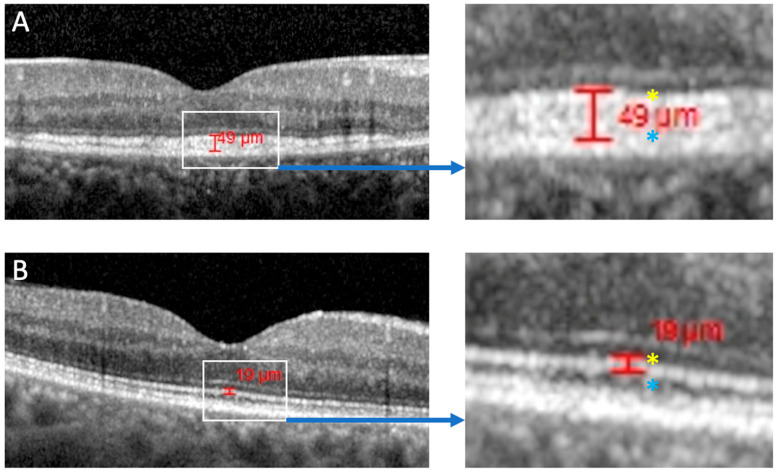
Optical coherence tomography image showing measurement of the ellipsoid zone (EZ) in patient 27A (**A**) and a normal image of the EZ (**B**). The red marker demarcates the upper and lower aspects of the EZ. For the purpose of labelling, the EZ was measured from the beginning of the second hyper-reflective (yellow asterisk) band to the first hypo-reflective region (above the RPE) (blue asterisk). Two image formats are available on the HEYEX2 software. The two measurements were performed on (1) a micrometre image modality for accuracy (as per guidance on HEYEX2), but representative images are shown in (2) pixel format to illustrate the structures more clearly.

OCT images were reviewed to identify the four outer retinal hyper-reflective bands [50]: band 1 represents the external limiting membrane, band 2 the ellipsoid zone (EZ), band 3 the interdigitation zone, and band 4 the retinal pigment epithelium (RPE)/Bruch’s complex. The thickness of band 2 (EZ) was manually measured by S.A.K using the inbuilt Heidelberg caliper (HEYEX 2) on a foveal B-scan slice in a region of the retina that appeared disease-free. This measurement was performed from the beginning of the hyper-reflective band until the first sign of hypo-reflectivity below using a magnification of 400–800% (Figure 1).

### 2.3. Genetic Analysis

DNA was extracted from peripheral venous blood samples. The retrospective nature of this study meant that sequencing was performed according to the most appropriate method available at the Oxford Regional Genetics Laboratory at the time at which patients presented. Enrichment of the *PRPH2* gene was achieved using a customised HaloPlex enrichment system kit (Agilent Technologies, Santa Clara, CA, USA) designed to capture the coding exons and 10bp flanking introns [51]. Between 2019 and 2022, samples were prepared using Twist Human Core Exome. NGS was carried out using an Illumina MiSeq instrument (Illumina, San Diego, CA, USA) machine using a MiSeq v3 kit (Illumina, San Diego, CA, USA) as per the manufacturer’s instructions [51]. Multiplex ligation-dependent probe amplification (MLPA) analysis was also carried out to confirm exon 3 deletions in two patients. All single-nucleotide variant (SNV) findings were validated by Sanger sequencing. The detected variants were filtered based on their allele frequency in the Genome Aggregation Database (gnomAD, https://gnomad.broadinstitute.org, accessed on 28 June 2025). Variants with a minor allele frequency of over 0.1% were excluded. The LOVD (http://www.lovd.nl/PRPH2, accessed on 28 June 2025) and ClinVar (https://www.ncbi.nlm.nih.gov/clinvar/, accessed on 28 June 2025) databases were reviewed to investigate whether the detected variants were previously reported as pathogenic in other patients with *PRPH2* retinopathy. Variants were considered novel if they were not previously reported in these two databases.

Pathogenicity of the identified variants was predicted using in silico analysis. Missense variants were investigated using Polyphen2 (http://genetics.bwh.harvard.edu/pph2/) [52] (accessed on 28 June 2025), Sorting Intolerant from Tolerance (SIFT) (http://sift.jcvi.org/) [53] (accessed on 28 June 2025), and Mutation Taster (http://www.mutationtaster.org/) [54] (accessed on 28 June 2025). The combined annotation-dependent duplication (CADD) score was also calculated (https://cadd.gs.washington.edu) [55] (accessed on 28 June 2025). Splice defects were investigated using SpliceAI (https://spliceailookup.broadinstitute.org) [56] (accessed on 28 June 2025). All variants were screened for cryptic splicing effects. Variants were considered likely pathogenic if they were predicted to be disease-causing by at least two in silico analysis programmes. The evolutionary conservation of the novel missense variants was investigated by aligning amino acid sequences from different species using Clustal Omega (https://www.ebi.ac.uk/Tools/msa/clustalo/) [57] (accessed on 28 June 2025). Accession numbers were human (*Homo sapiens*) NP_000313.2; chimpanzee (*Pan troglodytes*) XP_001134673.1; cow (*Bos taurus*) NP_001159959.1; Rat (*Rattus norvegicus*) NP_037153.1; mouse (*Mus musculus*) NP_032964.1; chicken (*Gallus gallus*) NP_990369.1; and frog (*Xenopus tropicalis*) XP_002934729.1. Amino acid residues were considered highly conserved if they were preserved across all species or were only different in one species of either fish or reptiles, moderately conserved if they were different in from two to five species, and not conserved if they were different in more than five species or at least one primate [58]. The laboratory used the American College of Medical Genetics Criteria (ACMG) to grade the pathogenicity of all variants [59]. Only the final ACMG class (1–5) was available in the reports; individual evidence codes were not provided. For the purposes of this study, patients were considered to have a confirmed molecular diagnosis if they carried a variant of at least C3 (a variant of unknown significance) and had a strong phenotype consistent with *PRPH2*.

### 2.4. Statistical Analysis

Statistical analysis was carried out using Python v3.13.6. A *t*-test was used to compare continuous variables and a chi-square to analyse contingency tables. A *p*-value of less than 0.05 was considered statistically significant. The yearly rate of change in BCVA for patients with PD/AVMD and PSPD phenotypes was analysed using a simple linear regression. Separate analyses were performed for right and left eyes. The independent variable was follow-up time in years, and the dependent variable was the change in BCVA (logMAR). The slope of the regression line was used to calculate the average yearly change in BCVA.

## 3. Results

### 3.1. Clinical Findings

A total of 36 patients (14 male, 22 female) with *PRPH2* retinopathy were identified from 33 unrelated families. Ethnically, 34 patients were of European descent, one patient of South Asian descent (patient 29), and one patient of Caribbean descent (patient 2). Mean age at baseline examination was 54.6 ± 14.4 (range 26–86) years. Mean self-reported age of symptom onset was 44.0 ± 11.25 years. Four patients were asymptomatic and were incidentally noted to have retinal changes (baseline ages when retinal features were identified ranged from 39–66 years). An autosomal dominant inheritance pattern was reported by 17 families, and 15 cases were sporadic. Family history was not recorded for one case.

### 3.2. Phenotype on FAF and OCT Imaging

**Figure 2:**
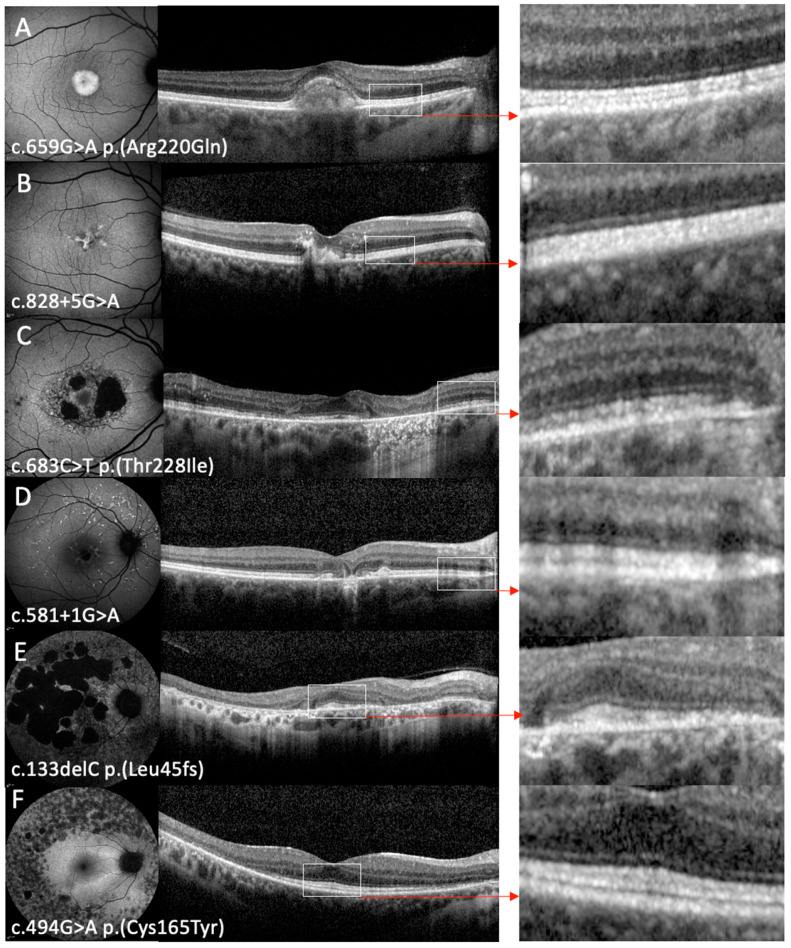
Fundus autofluorescence and optical coherence imaging showing the different phenotypes associated with variants in *PRPH2*. The FAF image is shown in the left panel, the OCT image is shown in the middle panel, and a magnified region of relatively uninvolved retinal tissue is shown in the right panel. All eyes shown are right eyes. (**A**) Adult vitelliform macular dystrophy phenotype in patient 13A, characterised by a circular region of raised signal from the vitelliform lesion on FAF imaging and a foveal vitelliform change on OCT imaging. (**B**) Pattern dystrophy phenotype in patient 2 characterised by butterfly-shaped flecks with raised signal on FAF imaging and foveal outer retinal and retinal pigment epithelial disruption on OCT imaging. (**C**) Mottled perifoveal AF with patches of retinal atrophy that spare the fovea on FAF and corresponding foveal-sparing macular atrophy on OCT imaging in patient 33. (**D**) Pseudo-Stargardt pattern dystrophy (PSPD) in patient 18 characterised by central macular atrophy and flecks in the posterior pole on FAF imaging and central macular atrophy on OCT imaging. (**E**) A more severe PSPD phenotype in patient 31 with coalescing patches of retinal atrophy associated with a heterogeneous background AF with relative peripapillary sparing on FAF imaging, and with a relatively spared fovea on OCT imaging. (**F**) Retinitis pigmentosa phenotype in patient 32 characterised by retinal atrophy in the mid periphery and the presence of a ring of raised signal around the macular region on FAF imaging and central retinal preservation on OCT imaging. The magnified panels on the right (**A**–**F**) illustrate EZ thickening and hyper-reflectivity across the different phenotypes.

The phenotypes observed in this cohort included PSPD in 17/36 patients (47.2%), AVMD in 8/36 (22.2%), PD in 9/36 (25%), RP in 1/36 (2.8%) patients, and an atypical phenotype characterised by patches of macular atrophy associated with mottled perifoveal autofluorescence (AF) in 1/36 (2.8%). Representative cases for these phenotypes are presented in Figure 2. Patient 16 had a different phenotype in each eye: AVMD in the right and PD in the left. Patient 9B progressed from a PD phenotype to a PSPD phenotype over the follow-up period. Five patients with PSPD had a heterogeneous background AF, indicative of more severe disease. OCT images were important for differentiating PD from AVMD in four patients who seemingly had a PD phenotype on the FAF images. One patient with AVMD (patient 18) had a bilateral CNV, which was treated with ranibizumab. Genotypes and clinical data are summarised in Table 1.

**Figure 3:**
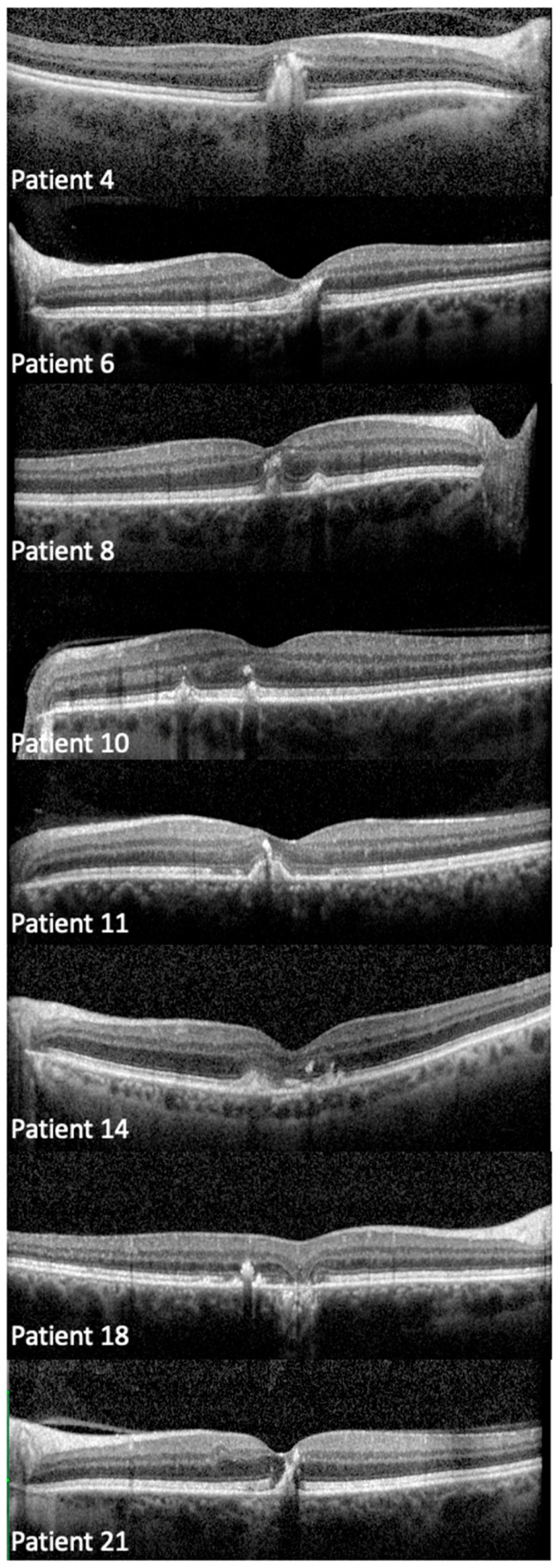
Representative optical coherence tomography images showing areas of increased retinal hyperreflectivity in the outer and inner retinal layers in eyes with *PRPH2* retinopathy.

Qualitatively, the EZ appeared thickened in 34 patients (see right panel of Figure 1). Mean EZ thickness was 44.3 ± 11.3 µm OD and 42.7 ± 11.6 µm OS, and was not significantly different between patients with PD/AVMD and those with PSPD in either right (45.2 ± 12.2 vs. 44.6 ± 10.5, *p* = 0.89) or left eyes (41.9 ± 11.9 vs. 44.3 ± 11.4, *p* = 0.56). Areas of hyper-reflectivity within the outer and inner retinal layers were observed in 20/36 (55.6%) patients with OCT imaging (Figure 3).

**Figure 4:**
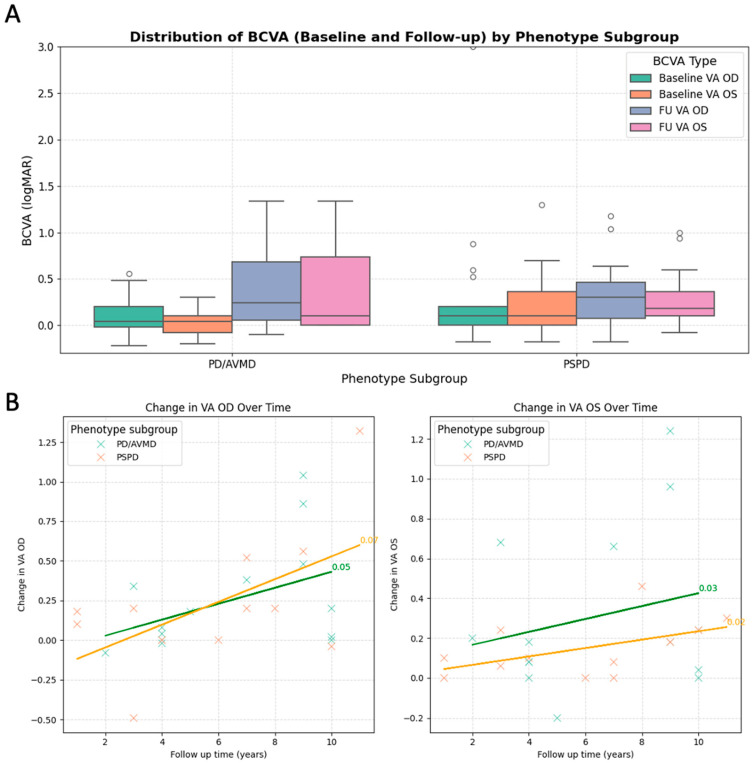
Visual acuities across the different *PRPH2* retinopathy phenotype subgroups. (**A**) Bar charts showing baseline and follow-up best corrected visual acuities (BCVAs) in right and left eyes. (**B**) Scatter plot showing the change in BCVA over time (years) for patients with PD/AVMD and PSPD phenotypes, for both right and left eyes. The gradient of the lines of best fit was calculated using simple linear regression and represents the average yearly change in BCVA. AVMD adult vitelliform macular dystrophy, BCVA best corrected visual acuity, PD pattern dystrophy, OD right eye, OS left eye.

The clinical characteristics for each phenotypic group are summarised in Table 2. For purposes of comparison, patients with PD and AVMD were grouped together and compared to those with PSPD. Age of onset was defined as that of the first self-reported symptom onset or when retinal features were first noted in asymptomatic patients. The age of onset and BCVAs in right eyes (Figure 4A) were not significantly different between patients with PD/AVMD and those with PSPD (*t*-test, *p* > 0.05). Mean baseline BCVAs were 0.20 ± 0.54 logMAR OD and 0.14 ± 0.29logMAR OS, and, at final follow-up, mean BCVAs were 0.33 ± 0.40 logMAR OD and 0.32 ± 0.40 logMAR OS. Mean follow-up duration was 6.0 ± 3.2 years (range 1–11 years). In all 36 patients, mean BCVAs were not significantly different between right and left eyes at baseline (paired *t*-test, *p* = 0.39) and final follow-up (paired *t*-test, *p* = 0.83). A linear regression model was used to assess whether the yearly change (gradient) in BCVA differed between PD/AVMD and PSPD, and this did not show a significant difference in both right (0.05 vs. 0.07 logMAR/year, *p* = 0.787) and left eyes (0.03 vs. 0.02 logMAR/year, *p* = 0.712) (Figure 4B). Patients with PSPD were significantly older than those with PD/AVMD (60.8 ± 11.8 vs. 49.2 ± 15.1 years, *t*-test, *p* = 0.018). BCVA was highest in the RP phenotype, which was not unexpected because of preserved macular structures in this group.

### 3.3. Genetic Characteristics

**Figure 5:**
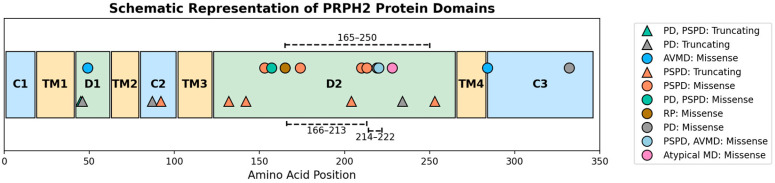
Schematic representation of PRPH2 protein and the detected variants.

We identified 26 different variants in *PRPH2,* of which eight were novel (Table 3). The 26 different variants included: 13 missense, 6 frameshift, 3 nonsense, 2 splice site, one seemingly synonymous variant that was predicted to affect splicing, and one exon deletion. Correlation between variants and their location along the protein revealed that 15/26 (57.5%) involved the D2 loop, 3/26 (11.5%) involved the D1 loop, 2/26 (7.7%) involved the C2 domain, and 2/26 (7.7%) involved the C3 domain (Figure 5).

The c.659G>A p.(Arg220Gln) variant was identified in 4/36 (11.1%) patients, making it the most frequently detected variant in our cohort. Genotype–phenotype correlation revealed that the D2 loop was a mutation hotspot that contained missense and truncating variants. Variants in the D2 loop were associated with significant phenotypic variability that included PD, AVMD, PSPD, RP, and an atypical macular dystrophy phenotype. The only patient with RP in our cohort carried the c.494G>A p.(Cys165Tyr) variant. The following variants were associated with more than one phenotype: c.133delC p.(Leu45fs), c.394delC p.(Gln132*), c.469G>A p.(Asp157Asn), c.612C>G p.(204*), and c.659G>A p.(Arg220Gln). There was no significant difference in the number of patients carrying truncating/splice site variants in patients with AVMD/PD and those with PSPD (Fisher’s exact test, *p* = 0.73).

**Figure 6:**
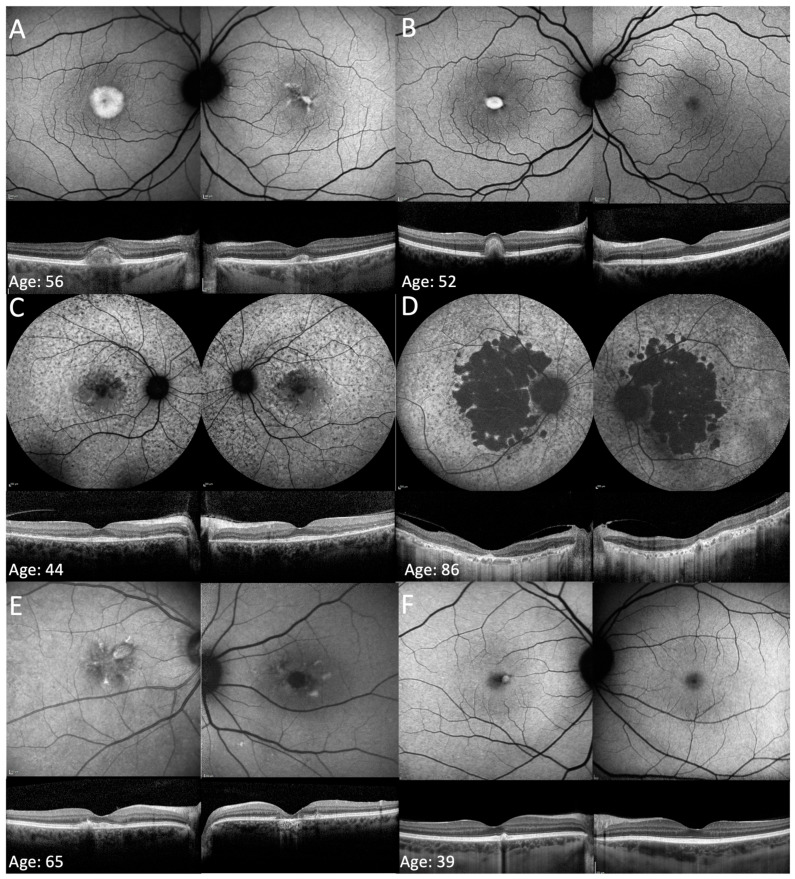
Retinal imaging in relatives with *PRPH2* retinopathy. (**A**,**B**) FAF imaging showing AVMD that is more significant in the right eye in siblings patient 13A (**A**) and patient 13B (**B**). (**C**,**D**) FAF and OCT imaging show a PSPD phenotype that spares the fovea in patient 27A, whilst her mother, patient 27B (**D**), had a more advanced phenotype with larger coalescing areas of atrophy. (**E**,**F**) FAF and OCT imaging showed PD phenotype in patient 9B, while her daughter, patient 9A, only had a perifoveal fleck in the right eye and significant thickening of band 2 in both eyes.

Intrafamilial comparisons for relatives carrying the same variants were possible in three families. Patients 13A and 13B, who were siblings with AVMD, carried the c.659G>A p.(Arg220Gln) variant and showed the same phenotype (Figure 6A,B). Intrafamilial variability was observed between patient 27A, who had a PSPD phenotype aged 44, and her mother (patient 27B), who had a PSPD phenotype aged 83 (c.612C>G p.(Tyr204*)), which could be attributed to a difference in disease duration (Figure 6C,D). Patient 9B initially had a PD phenotype at baseline, aged 65 years, and progressed to PSPD by age 74, whilst her daughter (patient 9A) had an early PD phenotype aged 39 (c.469G>A p.(Asp157Asn)) (Figure 6E,F).

**Figure 7:**
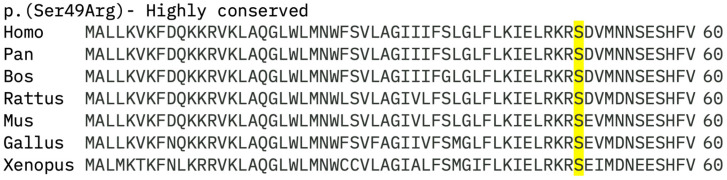
Sequence alignment of PRPH2 across species for novel missense variants. Assessment of evolutionary conservation of a novel missense PRPH2 variant that was shown to be conserved across all different species.

Of the novel variants identified, there were four frameshift mutations (two deletions, one insertion, and one duplication), two missense variants, a splice site variant, and one seemingly synonymous variant. The truncating variants were considered likely pathogenic as they are expected to undergo nonsense-mediated decay (NMD). The c.828G>A seemingly synonymous variant was classified as a variant of unknown significance (VUS) as it was predicted to cause a splicing defect by Splice AI and also had a CADD score of 25.3. The c.828+5G>A variant was predicted to cause a significant splicing defect by SpliceAI and also had a CADD score of 33. The c.147C>G p.(Ser49Arg) variant was only predicted to be pathogenic by Mutation Taster but was conserved across all different species (Figure 7), and likely pathogenic variants were previously reported on LOVD in the nearby Arg48 and Asp50 amino acid residues. This substitution results in a change from the neutral charged, small, and polar serine to the larger, polar, and bulky side chain containing arginine, which could potentially affect protein folding. The c.520T>C p.(Trp174Arg) variant was predicted to be pathogenic by Mutation Taster and SIFT. This variant results in the same amino acid change as the previously reported likely pathogenic c.520T>A p.(Trp174Arg).

## 4. Discussion

This study provides a comprehensive analysis of genotype–phenotype correlations in *PRPH2* retinopathy from a cohort of 36 patients from the Oxford Eye Hospital. Our findings highlight the significant phenotypic variability and complex nature of this condition and contribute valuable insights to the current understanding of *PRPH2* retinopathy.

Symptom onset occurred in the fifth decade or later in 75.9% of our cohort, which is consistent with previous reports [3,40]. The one patient with RP in our cohort reported the earliest symptom onset, which supports previous findings that RP patients manifest symptoms earlier, within the third-fifth [37] decade, compared to other *PRPH2* retinopathy phenotypes [3,17,18,40,60,61]. Mean BCVAs were 0.20 logMAR OD and 0.14 logMAR OS at baseline and 0.33 logMAR OD and 0.32 logMAR OS after a mean follow-up of 6.0 years. These were consistent with recently reported median BCVAs of 0.18 logMAR in a large international cohort of patients [40] and previous reports documenting BCVA in *PRPH2* retinopathy [3,34,35,48].

Our cohort exhibited a wide range of phenotypes, including PSPD (47.2%), AVMD (22.2%), PD (25%), atypical macular dystrophy (2.8%), and RP (2.8%). FAF imaging was highly valuable in delineating the different *PRPH2* retinopathy phenotypes, consistent with previous reports [48]. The predominance of the PSPD phenotype is consistent with recent findings by Heath-Jeffrey et al., who reported PSPD in 41% of their patients [40], and Reeves et al., who reported PSPD in 46.5% [34]. Of note, the PD and AVMD phenotypes that are generally considered the typical phenotypes in *PRPH2* accounted for 47.2% of our patients, whilst Heath Jeffrey et al. only noted this phenotype in 11% of their patients [40] and Reeves et al. reported these phenotypes in 28.9% of their *PRPH2* patients [34].

We report a lower proportion of RP and CACD cases compared to other published cohorts. The autosomal dominant retinitis pigmentosa (adRP) phenotype has been reported to account for 9.6–40% of *PRPH2* retinopathy cases [34,35,37,40,48]. Interestingly, Wang et al. recently reported that *PRPH2* variants were associated with adRP in 64% of their patients, making it more prevalent than macular dystrophy [62]. Moreover, variants in *PRPH2* are the cause of adRP in 8% of patients in Sweden [63], and are the second most prevalent cause of adRP in France, where it accounts for 10.3% of cases [39,64]. Similarly, the CACD phenotype has been reported to account for 7–28% [40,48], and this phenotype has been well characterised in the Dutch population by Boon et al. [10]. However, our cohort did not contain CACD patients. These phenotypic discrepancies could reflect population-specific differences. Similarly to Heath Jeffrey et al., our cohort did not reveal differences in sex distribution for the different phenotypes [40].

OCT images revealed significant loci of hyperreflectivity in 55.6% of our patients, which corresponded to hyperfluorescent fleck-like changes on FAF imaging. These foci had a particularly prominent appearance and often appeared to extend through multiple retinal layers—a feature that, to our knowledge, has not been specifically described in *PRPH2* retinopathy. Previously, Sparrow et al. suggested that hyperreflective foci in *PRPH2* retinopathy originate from the impaired photoreceptor cells, even in the absence of widespread lipofuscin accumulation [65]. Our findings support this hypothesis, and we propose that these foci likely result from *PRPH2* dysfunction, which impairs the formation and maintenance of photoreceptor outer segment discs and lamellae [3,23,24,25,26].

Abnormal ERG responses have been reported in 78.2–92% of *PRPH2* retinopathy patients [34,40], and cone and rod dysfunction has been reported in 50–68% of patients with a PSPD phenotype [40,66].

OCT imaging revealed a thickened and hyper-reflective EZ in 34/36 patients; this was not observed in two patients who both had AVMD. In two previous studies by Heath Jeffrey et al. [40,50], the EZ was also noted to be thickened. In our cohort, the EZ was significantly thicker at 43.9 µm OD and 42.2 µm OS compared to the 20.2 µm OD and 21.4 µm OS measurements reported by Heath Jeffrey et al. [50], and both studies show thickening compared to controls who had a mean EZ thickness of 17.0 µm reported by Heath Jeffrey et al. [50].

OCT Band 2 (see Figure 1) is thought to represent the mitochondria-rich EZ layer of the inner segment or the IS/OS junction [67]. It is possible, therefore, that the increased height of the EZ could be either due to thickening of the EZ itself, a greater reflectivity at the IS/OS junction, or fusion of the EZ with the interdigitation zone [50,68]. Heath Jeffrey et al. have suggested that this thickening may result from an accumulation of light-scattering subcellular structures within the IS, thus making band 2 thicker and potentially fusing bands 2 and 3. In addition, *PRPH2* mutant mice have been shown to have elongated, disorganised photoreceptor discs with compacted open discs and swollen mitochondria in the EZ [69]. We propose that the EZ thickness could be a useful biomarker for distinguishing *PRPH2* retinopathy from other macular dystrophies.

We identified 26 different variants in *PRPH2*, including 8 novel variants. In previous studies, the majority of variants were located in the D2 loop (up to 82%), which is consistent with our findings [3,34,35,40,70]. However, in our study, 43% of variants were located in the other domains of PRPH2. The p.(Arg172Trp) variant, which is frequently reported in the literature [3,10,71], was not detected in our cohort despite it being previously proposed to be a founder mutation in the British population [71,72].

Previously, loss-of-function *PRPH2* variants have been proposed to cause a rod-dominant phenotype due to haploinsufficiency [25,73,74,75,76], whilst gain-of-function variants result in a cone-dominant phenotype [76,77,78]. However, we did not identify any clear genotype–phenotype correlation in our cohort, which is similar to the Japanese cohort described by Oishi et al. [35]. There was phenotypic variability between unrelated patients carrying the same *PRPH2* variant, consistent with previous reports in the literature [15,48]. The most frequently detected variant in our cohort, c.659G>A p.(Arg220Gln), was associated with PSPD and AVMD phenotypes. This is in contrast to other studies where cone–rod dystrophy, PSPD, and RP were linked to variants in exon 1, while PD was linked to variants in exon 2 [34]. Heath Jeffrey et al. recently proposed that CACD is caused by specific missense variants, as none of their CACD patients carried truncating variants [40], and the p.(Arg172Trp) variant associated with macular dystrophy reported by Downes et al. is consistent with this [71,79]. By contrast, Bianco et al. reported that loss-of-function variants result in PD [61], while Peeters et al. [36] reported specific missense variants that were associated with adRP and PD phenotypes, thus highlighting the complexity of predicting phenotypes based on genotypes in *PRPH2*.

Of interest, our patient with an atypical macular dystrophy phenotype was heterozygous for c.683C>T p.(Thr228Ile). The same missense variant was identified by Heath Jeffrey et al. in patients with similar phenotypic features to our patient, which was described in their publication as CACD [40]. This variant has been previously linked to PD in two studies, but limited phenotypic information was available in the publications [11,34].

We did not find a significant difference in the proportion of patients with truncating variants in patients with PD, AVMD, and PSPD, which is consistent with Heath Jeffrey et al.’s observation that truncating variants were associated with variable phenotypes [40].

Phenotypic comparisons between affected family members carrying the same variant were possible in three families. We noted milder phenotypes in offspring compared to their parents, likely related to differences in disease duration. The two siblings in the same family with an AVMD phenotype (c.659G>A p.(Arg220Gln)) did not exhibit a significant phenotypic difference. Similarly, Sodi et al. reported two sisters with butterfly-shaped pattern dystrophy who had similar phenotypes and also had this variant [80]. Khan et al. reported this variant in a consanguineous family where both parents were asymptomatic. However, their FAF imaging showed central areas of increased AF, and the mother had a point of increased AF nasal to the fovea, while their son, who was significantly more affected and homozygous for this variant, had an ovoid ring of raised AF with points of increased AF temporal to the ring [81].

Other variants in *PRPH2* have shown significant intrafamilial variability with RP, PD, and PSPD with the same variant [9,14,15,48,66,82,83,84]. The p.(Arg172Trp) variant had been reported to have a consistent intra- and interfamilial phenotype characterised by central retinal changes [34,71,72,85]. Incomplete penetrance is common in association with *PRPH2* mutations [3,11,12,13,17,40,86]. One potential explanation for phenotypic differences is the presence of genetic modifiers, as demonstrated by digenic inheritance of mutations in *PRPH2* and *ROM1* resulting in an autosomal dominant RP phenotype [17,18,87]. *ABCA4* and *RPE65* have also been proposed as genetic modifiers that result in a more severe phenotype in *PRPH2* retinopathy [13,61,87,88].

Previously, Coco-Martin et al., Poloschek et al., and Bianco et al. reported that the co-inheritance of *PRPH2* and *ABCA4* results in a more severe phenotype [13,61,87]. However, it is important to be aware that patients may carry variants in multiple IRD genes without any clinical consequences when interpreting results on digenic or trigenic inheritance. Hanany et al. proposed that 36% of the world population is healthy carriers of at least one IRD variant [89], meaning that patients may carry variants in multiple IRD genes without any clinical consequences. Detecting more than one IRD gene poses a significant challenge to providing a molecular diagnosis and determining whether a variant has any modifying effects on other detected variants, and these dilemmas will be increasingly encountered as whole-genome sequencing becomes more available. Interestingly, Shankar et al. reported that *PRPH2 trans* modifiers can influence the phenotype in *PRPH2* retinopathy as they found that patients with the Gln304-Lys310-Asp338 (which is usually classified as a polymorphism) haplotype in *trans* with the *PRPH2* mutation were more likely to have severe phenotypes (CRD, RP, CACD) rather than PD [9]. Moreover, the effect of environmental factors on the phenotype remains to be explored as their role is unknown in *PRPH2* retinopathy [3,70,90].

The significant phenotypic variability in *PRPH2* retinopathy highlights the importance of furthering our understanding of PRPH2 function in rod and cone photoreceptors through cellular and animal models, as well as natural-history studies in well-characterised patient cohorts. Such studies will help understand potential disease mechanisms and guide therapeutic trial design. In particular, different *PRPH2* variants can act through loss of function (LoF) or gain/dominant-negative mechanisms. This means that mechanism-matched strategies may be required [91]. In preclinical studies, treatment of LoF by delivering a wild-type gene copy using an adeno-associated virus (AAV) has been shown to improve ERG amplitudes and outer-segment structure in mouse models, but this did not reduce the rate of photoreceptor loss [92]. By contrast, treatment of gain-of-function mutations would require knockdown of the mutant allele followed by replacement with a wild-type copy [93]. These differences imply that future trials should stratify cohorts by genotype and phenotype.

This study had several limitations. Firstly, the retrospective design of this study meant that there were instances of incomplete data, particularly in terms of clinical follow-up. Secondly, in silico analysis was used to investigate the pathogenicity of *PRPH2* variants, and a lack of in vitro functional studies limits our ability to confirm the functional effects and pathogenicity of the detected variants. Thirdly, whole genome sequencing was not available in routine use when the genetic testing in these patients was performed.

## 5. Conclusions

In conclusion, this study provides a comprehensive analysis of *PRPH2* retinopathies in a small cohort. Our findings emphasise the significant phenotypic variability and complex genotype–phenotype correlation in this disease, which is consistent with the literature. This highlights the need for caution when counselling families regarding prognosis. Thickening of the ellipsoid zone, as noted in the *PRPH2* retinopathy patients described in this study and reported in one previous study, could be a useful biomarker to distinguish *PRPH2* retinopathies from other phenocopies. Histopathological correlation with high-resolution OCT imaging would enable further insights into the pathophysiology of the development of the EZ thickening. The identification of novel variants reported herein expands the known genetic spectrum of *PRPH2*, contributing to improved molecular diagnosis and understanding of this complex retinal disease.

## Figures and Tables

**Table 1:** Summary of genotypes and phenotypes of patients with *PRPH2* retinopathy. AD autosomal dominant, AF autofluorescence, AMD age-related macular degeneration, asym asymptomatic, AVMD adult vitelliform macular dystrophy, VA best corrected visual acuity, F Female, FU follow-up, M Male, NR not recorded, OD right eye, ONL outer nuclear layer, OS left eye, PD pattern dystrophy, PSPD pseudostargardt pattern dystrophy, RP retinitis pigmentosa.

Case ID	Variant	Protein	Sex	Baseline Age	Onset	Symptoms	Inheritance	Baseline VA OD	Baseline VA OS	FU VA OD	FU VA OS	FU Time	FAF Phenotype	OCT Phenotype
1	c.656C>G	p.(Pro219Arg)	F	26	24	Visual distortion	AD	−0.02	−0.2	−0.10	0.00	2	PD	PD
2	c.828+5G>A		F	41	41	Slightly blurred vision at night	Sporadic	0.04	0.02	0.24	0.02	10	PD	PD
3	c.995T>A	p.(Val332Glu)	F	49	NR	NR	Sporadic	0.08	−0.14	0.10	0.10	10	PD	PD
4	c.259_266del	p.(Asp87Glnfs*87)	F	52	asym	asymptomatic	Sporadic	−0.10	0.14	0.24	0.82	3	PD	PD
5	c.629C>G	p.(Pro210Arg)	F	36	26	Photosensitivity and reduced night vision	AD	−0.22	0.00	NA	NA	0	PD	PD
6	c.700dup	p.(Tyr234fs)	F	43	43	Blurred vision	Sporadic	0.20	0.10	NA	NA	0	PD	PD
7	c.136C>T	p.(Arg46*)	F	41	41	Photosensitivity and delayed dark adaptation	AD	−0.04	0.04	1	1	9	PD	PD
8	c.133delC	p.(Leu45fs)	F	47	NR	NR	AD	0.10	0.06	0.1	0.1	10	PD	PD
9A	c.469G>A	p.(Asp157Asn)	F	39	asym	Asymptomatic	AD (mother 9B)	−0.08	−0.08	0.00	0.00	4	PD	PD
9B	c.469G>A	p.(Asp157Asn)	F	65	NR	NR	AD (daughter 9A)	0.20	0.54	0.40	1.00	8	PD->PSPD	Macular atrophy + flecks
10	exon 3 deletion		M	47	38	NR	Sporadic (AMD)	0.02	−0.08	0	0	4	PD	AVMD
11	c.828G>A	No AA change	M	45	45	Difficulty reading in low light	Sporadic	0.12	0.3	0.3	0.1	5	AVMD	AVMD
12	exon 3 deletion		M	46	NR	NR	Sporadic (AMD-mother)	0.02	−0.10	NA	NA	0	Atypical PD	Foveal EZ disruption OD and minimal AVMD OS
13A	c.659G>A	p.(Arg220Gln)	F	56	50	Visual distortion	AD (sister 13B)	0.56	0.30	0.94	0.96	7	AVMD OD PD → AVMD OS	AVMD
13B	c.659G>A	p.(Arg220Gln)	F	52	52	Blurred vision	AD (sister 13A)	0.30	0.10	0.78	0.28	9	AVMD	AVMD
14	c.850C>T	p.(Arg284Cys)	M	85	NR	NR	NR	0.40	0.30	0.40	0.48	4	PD	AVMD
15	c.147C>G	p.(Ser49Arg)	F	85	NR	Blurred vision	Sporadic	0.48	0.1	1.34	1.34	9	PD	AVMD + bilateral CNV
16	c.658C>T	p.(Arg220Trp)	M	47	47	NR	Sporadic	0	0	0.04	0	4	AVMD OD PD OS	AVMD OD PD OS
17	c.457A>G	p.(Lys153Glu)	F	52	asym	Asymptomatic	AD	0.1	0	0.1	0.1	4	PSPD	Flecks
18	c.581+1G>A	Splicing	M	52	52	Central visual distortion and delayed dark adaptation	Sporadic	−0.08	−0.06	NA	NA	0	PSPD	Macular atrophy + flecks
19	c.520T>C	p.(Trp174Arg)	M	50	58	Photosensitivity and blurred vision	AD	−0.14	0.36	−0.18	0.60	10	PSPD	Macular atrophy + spared fovea + flecks
20	c.394del	p.(Gln132fs)	F	50	50	Blurred vision, central scotomas, delayed dark adaptation, photosensitivity, and reduced night vision	AD	0.1	0.7	0.30	0.94	3	PSPD	Macular atrophy + foveal sparing
21	c.394del	p.(Gln132fs)	F	50	40	Blurred vision	AD	0.1	0.3	NR	NR	0	PSPD	Foveal atrophy + hyper-reflective material extending into ONL
22	c.756dup	p.(Ala253fs)	F	62	22	Blurred vision and metamorphopsia	Sporadic	0.88	0.04	0.39	0.1	3	PSPD	Macular atrophy OD + flecks OU
23	c.659G>A	p.(Arg220Gln)	M	78	NR	NR	AD	0.1	0.6	NA	NA	0	PSPD	Flecks
24	c.272_273insAT	p.(Ala92fs)	F	69	55	Photophobia and delayed dark adaptation	AD	0.52	0.22	1.04	0.22	7	PSPD + heterogenous background AF	Macular atrophy
25	c.638G>A	p.(Cys213Tyr)	M	52	52	Visual distortion	Sporadic	−0.18	−0.18	−0.08	−0.08	1	PSPD	Flecks
26	c.424delC	p.(Arg142fs)	M	78	30	Blurred vision	AD	0.60	0.30	NA	NA	0	PSPD	Macular atrophy
27A	c.612C>G	p.(Tyr204*)	F	44	44	Delayed dark adaptation, constricted visual fields and photosensitivity	AD (mother 27B)	0	0.1	0	0.1	6	PSPD + heterogenous background AF	Macular atrophy + foveal sparing
27B	c.612C>G	p.(Tyr204*)	F	86	50	Blurred vision	AD (daughter 27A)	HM	1.3	NA	NA	0	PSPD + heterogenous background AF	Macular atrophy
28	c.629C>G	p.(Pro210Arg)	M	56	54	Blurred vision and delayed dark adaptation	Sporadic	−0.14	−0.12	1.18	0.18	11	PSPD	Flecks + vitelliform lesion
29	c.659G>A	p.(Arg220Gln)	M	66	asym	asymptomatic	AD	0	0	0.18	0	1	PSPD	Macular atrophy + foveal sparing + flecks
30	c.394delC	p.(Gln132fs)	F	63	54	Distortion, delayed dark adaptation, photosensitivity, and central scotoma	AD	0.08	0.1	0.64	0.28	9	PSPD	Macular atrophy + foveal sparing OS
31	c.133del	p.(Leu45fs)	F	61	57	Delayed dark adaptation, photosensitivity, constriction of peripheral fields, and Charles Bonnet	Sporadic	0.1	0.1	0.3	0.18	7	PSPD + heterogenous background AF	Macular atrophy + foveal sparing
32	c.494G>A	p.(Cys165Tyr)	M	41	22	Nyctalopia and constricted visual fields	AD	0.00	0.00	0	0.00	6	RP	RP
33	c.683C>T	p.(Thr228Ile)	M	53	53	NR	Sporadic	0.08	0.00	−0.20	0.18	1	Atypical mottled perifoveal AF with patches of retinal atrophy	Macular atrophy + foveal sparing

**Table 2:** Clinical characteristics of AVMD/PD and PSPD. A Fisher’s exact test was used to investigate sex distribution. An unpaired *t*-test was used to investigate differences in age and BCVA between PD/AVMD and PSPD patients. AVMD adult vitelliform macular dystrophy, BCVA best corrected visual acuity, F female, PD pattern dystrophy, M male, OD right eye, OS left eye.

	PD/AVMD		PSPD		*p*=
**N=**	17		17		-
Sex	5 M	12 F	7 M	10 F	0.721
Age of onset (mean)	41.5 ± 9.1		49.1 ± 11.2		0.069
Age at baseline examination (mean)	49.2 ± 15.1		60.8 ± 11.8		0.018 *
Vision at baseline examination logMAR BCVA OD (mean)	0.11 ± 0.21		0.31 ± 0.75		0.300
Vision at baseline examination logMAR BCVA OS (mean)	0.05 ± 0.15		0.25 ± 0.37		0.045 *

Electrodiagnostic testing results were only available for 5/36 patients. The pattern electroretinogram (PERG), full field electroretinogram (ffERG), and electro-oculogram (EOG) were within normal limits in patient 2 and patient 9A, who both had PD. The three patients with PSPD all had abnormal cone and rod responses (patients 19, 24, and 31). Statistically significant results are denoted with *.

**Table 3:** Variant list with in silico analysis predictions and their observed phenotypes. AA amino acid, AVMD adult vitelliform macular dystrophy, ACMG American College of Medical Genetics and Genomics, N no, PD pattern dystrophy, PSPD pseudostargardt pattern dystrophy, Y yes. Diagnostic reports only provided the final ACMG class (1–5); individual evidence codes were not available. A SpliceAI score was calculated for all variants, 0 indicates no predicted splice effect.

Variant	Amino Acid	Mutation Type	Exon/Intron	Protein Domain	GnomAD	Mutation Taster	Polyphen-2	SIFT	CADD Score	SpliceAI Score	Novel	ACMG Class	Clinical Phenotype	Allele Count
c.133delC	p.(Leu45fs)	Frameshift	Ex 1	D1	1.24 × 10^−6^	Disease causing	-	-	-	0	Y	5	PD, PSPD	2
c.136C>T	p.(Arg46*)	Nonsense	Ex 1	D1	6.20 × 10^−6^	Disease causing	-	-	37	0	N	5	PD	1
c.147C>G	p.(Ser49Arg)	Missense	Ex 1	D1	1.24 × 10^−6^	Disease causing	benign	Tolerated	4.18	0	Y	3	AVMD	1
c.259_266del	p.(Asp87Glnfs*87)	Frameshift	Ex 1	C2	4.96 × 10^−6^	Disease causing	-	-	-	0	N	5	PD	1
c.272_273insAT	p.(Ala92fs)	Frameshift	Ex 1	C2	NR	Disease causing	-	-	-	0	Y	5	PSPD	1
c.394delc	p.(Gln132fs)	Nonsense	Ex 1	D2	8.05 × 10^−6^	-	-	-	-	0	N	5	PSPD	2
c.424delC	p.(Arg142fs)	Frameshift	Ex 1	D2	NR	Disease causing	-	-	-	0	Y	5	PSPD	1
c.457A>G	p.(Lys153Glu)	Missense	Ex 1	D2	NR	Disease causing	Probably damaging	Not tolerated	27.3	0	N	3	PSPD	1
c.469G>A	p.(Asp157Asn)	Missense	Ex 1	D2	NR	Disease causing	Probably damaging	Not tolerated	32	0	N	4	PD, PSPD	2
c.494G>A	p.(Cys165Tyr)	Missense	Ex 1	D2	NR	Disease causing	Probably damaging	Not tolerated	30	0	N	5	RP	1
c.520T>C	p.(Trp174Arg)	Missense	Ex 1	D2	6.19 × 10^−7^	Disease causing	Probably damaging	Not tolerated	29.2	0	Y	3	PSPD	1
c.581+1G>A		Splicing		-	6.20 × 10^−7^	-	-	-	29.3	0.99	N	5	PSPD	1
c.612C>G	p.(Tyr204*)	Nonsense	Ex 2	D2	6.20 × 10^−7^	Disease causing	-	-	40	0	N	5	PSPD	2
c.629C>G	p.(Pro210Arg)	Missense	Ex 2	D2	1.24 × 10^−6^	Disease causing	Probably damaging	Not tolerated	33	0	N	4	PD, PSPD	1
c.638G>A	p.(Cys213Tyr)	Missense	Ex 2	D2	4.96 × 10^−6^	Disease causing	Probably damaging	Not tolerated	33	0	N	4	PSPD	1
c.656C>G	p.(Pro219Arg)	Missense	Ex 2	D2	9.29 × 10^−6^	Disease causing	Probably damaging	Not tolerated	29.1	0	N	3	PD	1
c.658C>T	p.(Arg220Trp)	Missense	Ex 2	D2	4.96 × 10^−6^	Disease causing	Probably damaging	Not tolerated	25.2	0	N	4	PD, AVMD	1
c.659G>A	p.(Arg220Gln)	Missense	Ex 2	D2	5.58 × 10^−6^	Disease causing	Probably damaging	Not tolerated	32	0	N	3	PSPD, AVMD	4
c.683C>T	p.(Thr228Ile)	Missense	Ex 2	D2	5.33 × 10^−5^	Disease causing	Possibly damaging	Not tolerated	26.4	0	N	3	Atypical macular dystrophy	1
c.700dup	p.(Tyr234fs)	Frameshift	Ex 2	D2	1.24 × 10^−6^	-	-	-	-	0	N	5	PD	1
c.756dup	p.(Ala253fs)	Frameshift	Ex 2	D2	NR	Disease causing	-	-	-	0	Y	5	PSPD	1
c.828G>A	No AA change	Seemingly synonymous	Ex 2		-	Disease causing	-	-	25.3	0.91	Y	3	AVMD	1
c.850C>T	p.(Arg284Cys)	Missense	Ex 2	C3	2.48 × 10^−6^	Disease causing	Probably damaging	Not tolerated	34	0	N	3	AVMD	1
c.828+5G>A		Splicing			NR	-	-	-	33	0.79	Y	3	PD	1
c.995T>A	p.(Val332Glu)	Missense	Ex 3	C3	4.34 × 10^−6^	Disease causing	Probably damaging	Tolerated	25.6	0	N	3	PD	1
exon 3 deletion		Exon deletion	Ex 3	-	-	-	-	-	-	-	N	5	AVMD	2

## Data Availability

Dataset available on request from the authors.

## Abbreviations

The following abbreviations are used in this manuscript:

AF	Autofluorescence
adRP	Autosomal dominant retinitis pigmentosa
AVMD	Adult-onset vitelliform macular dystrophy
BCVA	Best corrected visual acuities
EZ	Ellipsoid zone
FAF	Fundus autofluorescence
IRD	Inherited retinal disease
LoF	Loss of function
OCT	Optical coherence tomography
NGS	Next-generation sequencing
NMD	Nonsense mediated decay
OD	Right eye
OS	Left eye
PD	Pattern dystrophy
PRPH2	Peripherin 2
PSPD	Pseudo-Stargardt pattern dystrophy
RDS	Retinal degeneration slow
RP	Retinitis pigmentosa
RPE	Retinal pigment epithelium
SNV	Single-nucleotide variant
VUS	Variant of unknown significance
